# Integrated Analyses Identify Key Molecules and Reveal the Potential Mechanism of miR-182-5p/FOXO1 Axis in Alcoholic Liver Disease

**DOI:** 10.3389/fmed.2021.767584

**Published:** 2021-12-07

**Authors:** Zhihua Zuo, Yiqin Li, Chuyi Zeng, Yuge Xi, Hualin Tao, Yongcan Guo

**Affiliations:** ^1^Department of Laboratory Medicine, The Affiliated Hospital of Southwest Medical University, Luzhou, China; ^2^Department of Clinical Laboratory, The Affiliated Traditional Chinese Medicine Hospital of Southwest Medical University, Luzhou, China

**Keywords:** alcoholic liver disease, key molecules, miR-182-5p, FOXO1, lipid metabolism

## Abstract

**Background:** Alcoholic liver disease (ALD) is one of the most common chronic liver diseases worldwide. However, the potential molecular mechanism in ALD development remains unclear. The objective of this work was to identify key molecules and demonstrate the underlying regulatory mechanisms.

**Methods:** RNA-seq datasets were obtained from Gene Expression Omnibus (GEO), and key molecules in ALD development were identified with bioinformatics analysis. Alcoholic liver disease mouse and cell models were constructed using Lieber-DeCarli diets and alcohol medium, respectively. Quantitative real-time PCR and Western blotting were conducted to confirm the differential expression level. Dual-luciferase reporter assays were performed to explore the targeting regulatory relationship. Overexpression and knockdown experiments were applied to reveal the potential molecular mechanism in ALD development.

**Results:** Between ALD patients and healthy controls, a total of 416 genes and 21 microRNAs (miRNAs) with significantly differential expression were screened. A comprehensive miRNA-mRNA network was established; within this network, the miR-182-5p/FOXO1 axis was considered a significant pathway in ALD lipid metabolism. Mouse and cell experiments validated that miR-182-5p was substantially higher in ALD than in normal livers, whereas the expression of FOXO1 was dramatically decreased by alcohol consumption (*P* < 0.05). Next, dual-luciferase reporter assays demonstrated that miR-182-5p directly targets the binding site of the FOXO1 3′UTR and inhibits its mRNA and protein expression. In addition, miR-182-5p was found to promote hepatic lipid accumulation via targeting the FOXO1 signaling pathway, and inhibition of the miR-182-5p/FOXO1 axis improved hepatic triglyceride (TG) deposition in ALD by regulating downstream genes involved in lipid metabolism.

**Conclusion:** In summary, key molecules were identified in ALD development and a comprehensive miRNA–mRNA network was established. Meanwhile, our results suggested that miR-182-5p significantly increases lipid accumulation in ALD by targeting FOXO1, thereby providing novel scientific insights and potential therapeutic targets for ALD.

## Introduction

Alcoholic liver disease (ALD), a chronic consequence of excessive long-term alcohol consumption, is among the most common chronic liver diseases. According to the World Health Organization, more than 3 million deaths were due to alcohol abuse in 2016 alone (1), and heavy drinking continues to be ubiquitous worldwide, thus causing a prevalence of ALD above 6% in Western developed countries (2). In the liver, abundant acetaldehyde and acetic acid are produced in alcohol catabolism, thus perturbing the redox balance and hepatic lipid metabolism by affecting biological enzyme activity, and leading to abnormal fat accumulation in the liver (3). Additionally, alcohol directly stimulates the activation of innate immune cells and the secretion of inflammatory cytokines (4). These pathologies are hallmarks of early-stage ALD, including alcoholic fatty liver disease (AFLD) and alcoholic hepatitis (AH); nevertheless, evidence has demonstrated that a lack of early and proper treatment for patients with these diseases markedly increases the risk of developing advanced stage ALD (liver fibrosis) and even alcoholic liver cirrhosis (5). Therefore, developing precisely targeted treatments can contribute to decreasing the burden of ALD among individuals and societies.

MicroRNAs (miRNAs), small non-coding RNAs 20–25 nucleotides in length, actively participate in the regulation of gene expression by targeting mRNA 3′UTR sites and promoting mRNA degradation and/or translational inhibition (6, 7). Accumulating evidence indicates that miRNAs play essential roles in liver-related diseases. For instance, miR-30b-5p promotes hepatocellular carcinoma (HCC) cell growth, proliferation, and metastasis by targeted inhibition of inositol polyphosphate phosphatase 1 expression (8). In ALD, over-expression of miR-203 decreases hepatic lipid accumulation by targeting the Lpin1 gene (Lipin1) (9). Silencing miR-21 decreases cytokine production and inflammatory responses in alcohol-induced hepatitis (10). However, hubs and comprehensive miRNA–mRNA regulatory networks have scarcely been reported. Importantly, the expression of several miRNAs, such as miR-182-5p, has been controversial in previous publications. miR-182-5p has been identified as a key regulator in the development and progression of ALD. Blaya has found that miR-182-5p is the most significantly upregulated miRNA in ALD and is associated with disease severity and liver injury (11). In contrast, Dolganiuc has reported that miR-182 is remarkedly down-regulated in Lieber-deCarli alcohol diet feeding compared to corresponding controls (12). In addition, the mechanism of miR-182-5p in ALD remains unclear.

In the present study, gene and miRNA expression profiles were used to screen differentially expressed signaling molecules in ALD through bioinformatics analyses. Next, genes targeted by miRNAs were predicted in comprehensive databases; notably, we combined multiple independent tools to enhance the accuracy and reliability of the results. Afterwards, a complex miRNA–mRNA network was established for ALD through intersection analysis, and R packages were used to conduct functional annotation analyses of hub genes. On the basis of our previous work and other related articles, the miR-182-5p/Forkhead Box Protein O1 (FOXO1) axis was identified and served as a case study for thorough investigation. Alcoholic liver disease mouse and cell models were constructed to examine the expression levels of miR-182-5p and FOXO1; dual-luciferase reporter assays were then used to explore their regulatory relationship; knockdown and overexpression experimental studies were performed to investigate the mechanism underlying the effects of the miR-182-5p/FOXO1 signaling pathway in lipid accumulation in ALD. Our results revealed a comprehensive miRNA–mRNA network and highlighted the potential of miR-182-5p in ALD development through experimental verification. Our findings provide new scientific insights and potential therapeutic targets for ALD.

## Materials and Methods

### Data Acquisition and Differential Expression Analysis

Three RNA-seq datasets (GSE28619, GSE143318, and GSE59492) were retrieved from Gene Expression Omnibus (GEO). GSE28619 included mRNA expression profiles of 15 ALD samples and seven normal liver samples, whereas GSE143318 included five and five, respectively. MicroRNAs expression data for 13 ALD tissues and six normal liver tissues were collected from GSE59492. The expression matrix was constructed with the R package affyPLM, and probe IDs were converted to gene symbols according to the corresponding platform annotation files. Gene expression values were averaged if matched with multiple IDs.

The original expression data were pre-processed with the robust multiarray average algorithm in R studio software, and the classical Bayesian algorithm was used to screen differentially expressed genes (DEGs) and differentially expressed miRNAs (DEMs) with the “limma” package. FDR (adj. *P*-value) < 0.05 and |log2 fold change (FC)| ≥1.5 were set as the cut-off criteria. Notably, the differential expression analysis between ALD and normal samples has been completed in GSE143318, which was viewed as a validation group of DEGs.

### Construction of a Protein–Protein Interaction Network and Functional Enrichment Analysis

Only common DEGs between GSE28619 and the validation group (GSE143318 and our previous work) were selected for further analysis. The protein–protein interaction (PPI) relationship of DEGs was mapped onto the Search Tool for the Retrieval of Interacting Genes (STRING, version 10.0, http://string.embl.de/). According to the default settings, the PPI network was constructed with a minimum required interaction score of 0.4, and disconnected genes were hidden. The results were visualized with Cytoscape 3.8.2 software. The R package clusterProfiler was used to perform the functional annotation analyses, comprising gene ontology (GO) enrichment and Kyoto encyclopedia of genes and genomes (KEGG) pathway analyses, which were depicted graphically with the “ggplot2” package.

### Target Gene Prediction and miRNA–mRNA Network Establishment

The prediction of DEM-target genes was achieved with two independent online databases: TargetScanHuman 7.2 (http://www.targetscan.org/vert_72/) and miRDB (http://mirdb.org/index.html). The fundamental criteria were based on the experiment, reference, and support type (functional miRNA–target interactions) to explore the most relevant miRNA–mRNA interactions. Afterwards, FunRich analysis between DEMs target genes and DEGs was performed to screen the overlapping genes, thereby establishing the miRNA–mRNA network and identifying hub genes and miRNAs of ALD.

### Animal and Cell Models of ALD

For the ALD model, C57BL/6 male mice, 6–8 week-old, each weighing 18–20 g, were purchased from SIPEIFU Biotechnology Ltd. (Beijing, China) and were housed in an animal facility with constant temperatures. The animal experimental procedures were approved by the Ethics Committees of Southwest Medical University (No.201809121). The mice were then randomly divided into ALD and control groups after acclimating to the laboratory conditions. Alcoholic liver disease mice were fed Lieber-DeCarli diets (Trophic Animal Feed High-tech Co., Ltd., China), and the model was constructed as follows: (1) adaptive feeding with an ordinary liquid diet for 3-days; (2) gradual addition of an alcohol liquid diet to the feed (9, 14, 19, 24, and 28% for 2 days, respectively); and (3) feeding with a 28% alcohol liquid diet for 26 days. The control mice were fed an ordinary liquid diet. Subsequently, the livers were quickly excised, and a portion of the tissues was fixed in 10% neutral-buffered formalin for pathological analysis; the remaining tissues and blood were stored at −80°C for further analyses.

Normal liver cell L02 line was obtained from the Laboratory Medicine of Chongqing Medical University (Chongqing, China) and cultured in RPMI 1640 (Gibco, United States) supplemented with 10% fetal bovine serum (Gibco, United States) and 100 U/ml Penicillin-Streptomycin Solution (Thermo Fisher Scientific, China). After reaching 50–60% confluence, L02 cells were stimulated with 100 mM alcohol medium for 48 h to construct the ALD cell model (9, 13). All cells were grown at 37°C in a humidified incubator containing 5% CO_2_.

### H&E Staining, Oil Red O Staining, and Testing of Biochemical Characteristics

H&E and Oil red O staining kits were purchased from Sangon Biotech Co., Ltd. (Shanghai, China). First, the mouse liver tissues were embedded in paraffin blocks, and L02 cells were fixed with 4% paraformaldehyde for 24 h and 60 min, respectively. Second, H&E and Oil red O staining was performed according to the manufacturers' protocols. The serum biochemical characteristics of the mice, such as alanine aminotransferase (ALT), aspartate aminotransferase (AST), triglycerides (TG), and total cholesterol (TC), were detected with a BS2000M automatic biochemical analyzer (Mindray, Shenzhen, China); The TG level of L02 cells was tested with a Tissue and cell TG assay kit (Applygen, Beijing, China). The absorbance was measured at 550 nm. Image pro plus software was applied to calculate the integrated optical density (IOD) of the oil-red O staining, thereby performing the quantitative analysis.

### miRNA Mimics, Inhibitor, Negative Control, and siRNA Transfection

To explore the importance of miRNA and the regulated relationship of miRNA–mRNA in ALD, we transfected L02 cells with 50 nM miRNA mimics, inhibitor, negative control (NC), and FOXO1 siRNA with Lipofectamine 2000 (Invitrogen, United States) and Opti-MEM (Gibco, United States), on the basis of the manufacturer's instructions. After 48 h of transfection, cells were collected for further analyses. The RNA oligo sequences were as follows: miRNA mimics: 5′-UUUGGCAAUGGUAGAACUCACACU-3′; miRNA inhibitor: 5′-AGUGUGAGUUCUACCAUUGCCAAA-3′; FOXO1 siRNA: 5′-UGACUUGGAUGGCAUGUUC-3′; NC: 5′-UUGUACUACACAAAAGUACUG-3′.

### RNA Isolation and Quantitative Real-Time PCR

Total RNA was extracted from mouse liver tissues and L02 cells with the Trizol method. According to the manufacturer's instructions, the cDNA for miRNAs was first synthesized with miRNA first strand cDNA synthesis (Sangon Biotech, Shanghai, China). For mRNA, a PrimeScript™ RT reagent Kit (TaKaRa, Shiga, Japan) was used. Then quantitative real-time PCR (RT-qPCR) was performed with 2 × SG Fast qPCR Master Mix (Low Rox) (Sangon Biotech, Shanghai, China) on Applied Biosystems 7500 Real-Time PCR Detection System (Applied Biosystems, United States) according to the manufacturer's instructions. SnRNA U6 and GAPDH were used as the internal controls for miRNA and mRNA, respectively. Primer sequences are shown in Table 1. The relative levels were calculated with the 2-^ΔΔ^Ct method, and all experiments were performed in triplicate and were repeated at least three times.

**Table 1:** Primer sequences of miRNA and mRNA used in the present work.

**Name**	**Forward primer**	**Reverse primer**
MiR-182-5p RT primer	GTCGTATCCAGTGCAGGGTCCGAGGTATTCGCACTGGATACGA CAGTGTG	
MiR-182-5p	GCGTTTGGCAATGGTAGAACT	AGTGCAGGGTCCGAGGTATT
snRNA U6 RT primer	GTCGTATCCAGTGCAGGGTCCGAGGTATTCGCACTGGATACGAC AAAATA	
snRNA U6	CTCGCTTCGGCAGCACA	AACGCTTCACGAATTTGCGT
FOXO1	AAACACCAGTTTGAATTCACCC	TCGACTTATTGTCCTGAAGTGT
SIRT1	AGACACGCTGGAACAGGTTG	CCTCGTACAGCTTCACAGTCA
ATGL	GCTCCACCAACATCCACGAG	TGCTTGCACATCTCTCGCAG
SREBP-1c	CCAGCGTCTACCATAGCCCT	GAAGCACCAAGGAGACGAGC
FASN	CCATCTACAACATCGACACCAG	CTTCCACACTATGCTCAGGTAG
CYP2E1	CCATCAAGGATAGGCAAGAGAT	ATTCAGGAAGTGTTCTGGCTTA
GAPDH	CGGAGTCAACGGATTTGGTCG TAT	AGCCTTCTCCATGGTGGTG AAGAC

*RT, reverse transcription; FOXO1, forkhead box protein O1; SIRT1, sirtuin 1; ATGL, adipose triglyceride lipase; SREBP-1c, sterol regulatory element-binding protein-1c; FASN, fatty acid synthase; CYP2E1, cytochrome P-450 2E1*.

### Dual-Luciferase Reporter Assay

The binding sites of the mRNA 3′UTR regions targeted by miRNAs were predicted with the TargetScan database, and the sequences, including wild type and mutant with spacer mutations, were then cloned and inserted into the luciferase reporter pmirGLO vector (Promega, WI, USA). Subsequently, 50 nM miRNA mimic, normal control, and 1 μg pmirGLO vector were co-transfected into HEK 293T cells. Cells were plated on 24-well plates, and harvested after 48 h of transfection. The relative activity of firefly luciferase was detected with the Dual-Glo luciferase assay kit (Beyotime, Shanghai, China).

### Western Blotting

Protein was extracted with Western Cell Lysis Buffer with 1% phenylmethyl sulfonyl fluoride (Sangon Biotech, Shanghai, China). Next, a modified BCA Protein Assay Kit (Sangon Biotech, Shanghai, China) was used to determine the protein concentration according to the absorbance at 562 nm. The 30 μg total protein was loaded onto an SDS polyacrylamide gel (Solarbio, Beijing, China), including 5% stacking gel and 8% resolving gel. After electrophoresis at 75 V for 30 min and 100 V for 60 min, protein was transferred to a 0.45 μm PVDF membrane (Millipore, Billerica, MA, United States) at 100 V for 2–4 h, blocked in 5% non-fat milk for 60 min, and incubated with primary antibodies for 12 h at 4°C (Abcam, UK) (FOXO1 69KD, 1:1,000, ab179450; sirtuin 1 (SIRT1) 110KD, 1:1,000, ab189494; adipose triglyceride lipase (ATGL) 55KD, 1:1,000, ab109251; sterol regulatory element-binding protein-1c (SREBP-1c) 127KD, 5 μg/ml, ab3259; fatty acid synthase (FASN) 273KD, 1:1,000, ab128856; GAPDH 37 KD, 1:10,000, ab181602); CYP2E1 (57KD, 1:2,000, 19937-1-AP) (Proteintech, UK), and the HRP conjugated secondary antibody (Beyotime, Shanghai, China) for 60 min. Subsequently, a BeyoECL Star kit (Beyotime, Shanghai, China) was used to conduct the western blotting detection, and the relative quantitative analyses were performed in Image J software.

### Statistical Analysis

All statistical analyses were performed in GraphPad Prism 8.0 and R studio 3.6.0. Quantitative data are represented with means ± standard deviation. Analysis of variance and Student's *t*-test were used to explore the differences among groups. *P*-values < 0.05 were considered statistically significant.

## Results

### Identification of DEGs and DEMs

**Figure 1:**
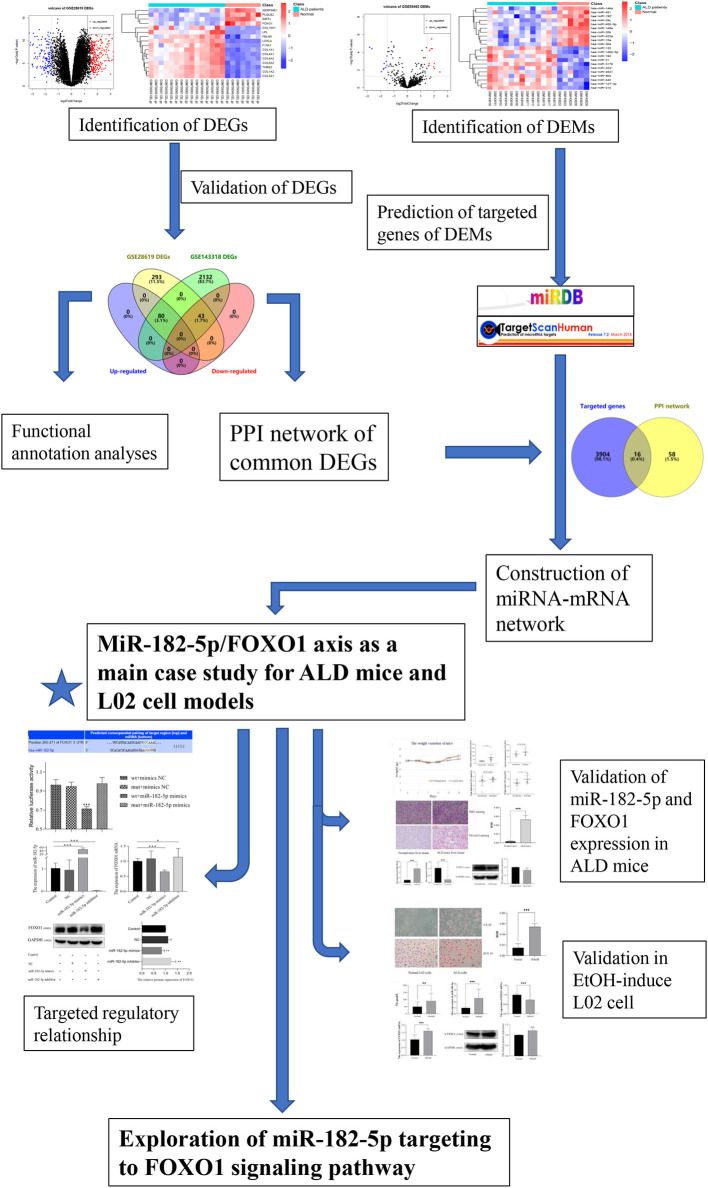
Flowchart of the current study. DEGs, differentially expressed genes; DEMs, differentially expressed miRNAs; ALD, alcoholic liver disease; PPI, protein–protein interaction.

**Figure 2:**
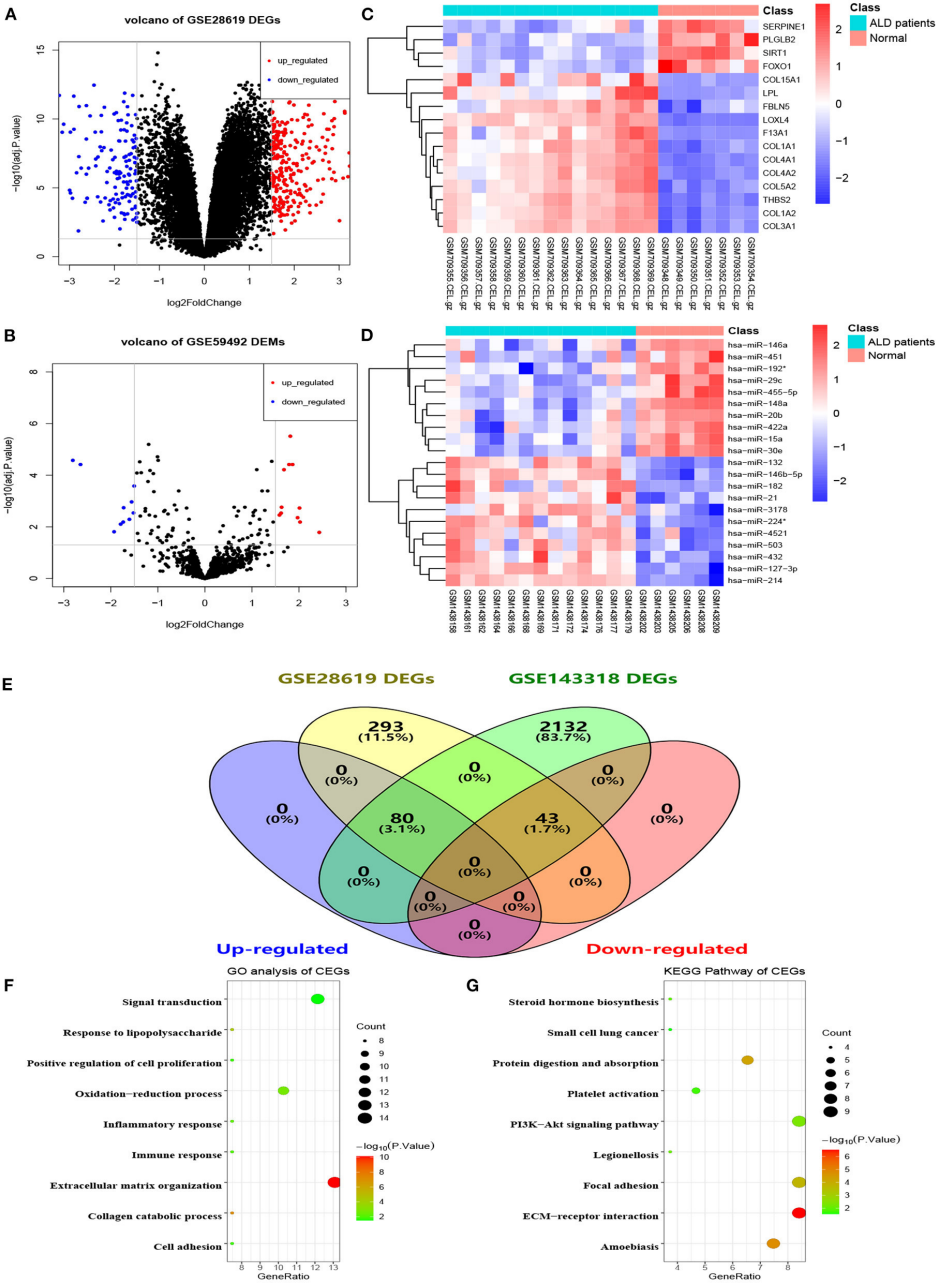
Identification of differentially expressed genes and miRNAs between patients with alcoholic liver disease and healthy controls. **(A)** Volcano plot of DEGs in GSE28619, **(B)** volcano plot of DEMs in GSE59492, **(C,D)** heatmaps of partial DEGs and DEMs, **(E)** common DEGs between GSE28619 and GSE143318, **(F)** GO enrichment analysis of CEGs, **(G)** KEGG pathway analysis of CEGs. DEGs, differentially expressed genes; DEMs, differentially expressed miRNAs; ALD, alcoholic liver disease; CEGs, common differentially expressed genes; GO, gene ontology; KEGG, kyoto encyclopedia of genes and genomes.

The flowchart of the current study is shown in Figure 1. On the basis of the defined criteria, a total of 416 DEGs and 21 DEMs were identified in GSE28619 and GSE59492 between ALD samples and normal liver samples, respectively. Volcano plots (Figures 2A,B) and heatmaps (Figures 2C,D) were constructed to reveal the details. In total, 123 DEGs were confirmed to be significantly differentially expressed in GSE143318 gene profiling (Figure 2E), including 80 up-regulated and 44 down-regulated DEGs. Gene ontology enrichment analysis (Figure 2F) further illustrated that common DEGs were highly involved in signal transduction, oxidation–reduction process, and extracellular matrix organization; Additionally, KEGG pathway analysis showed that the PI3K–Akt signaling pathway, focal adhesions, and ECM–receptor interaction were significantly associated with ALD development (Figure 2G).

### Construction of Protein–Protein Interaction and miRNA–mRNA Network

**Figure 3:**
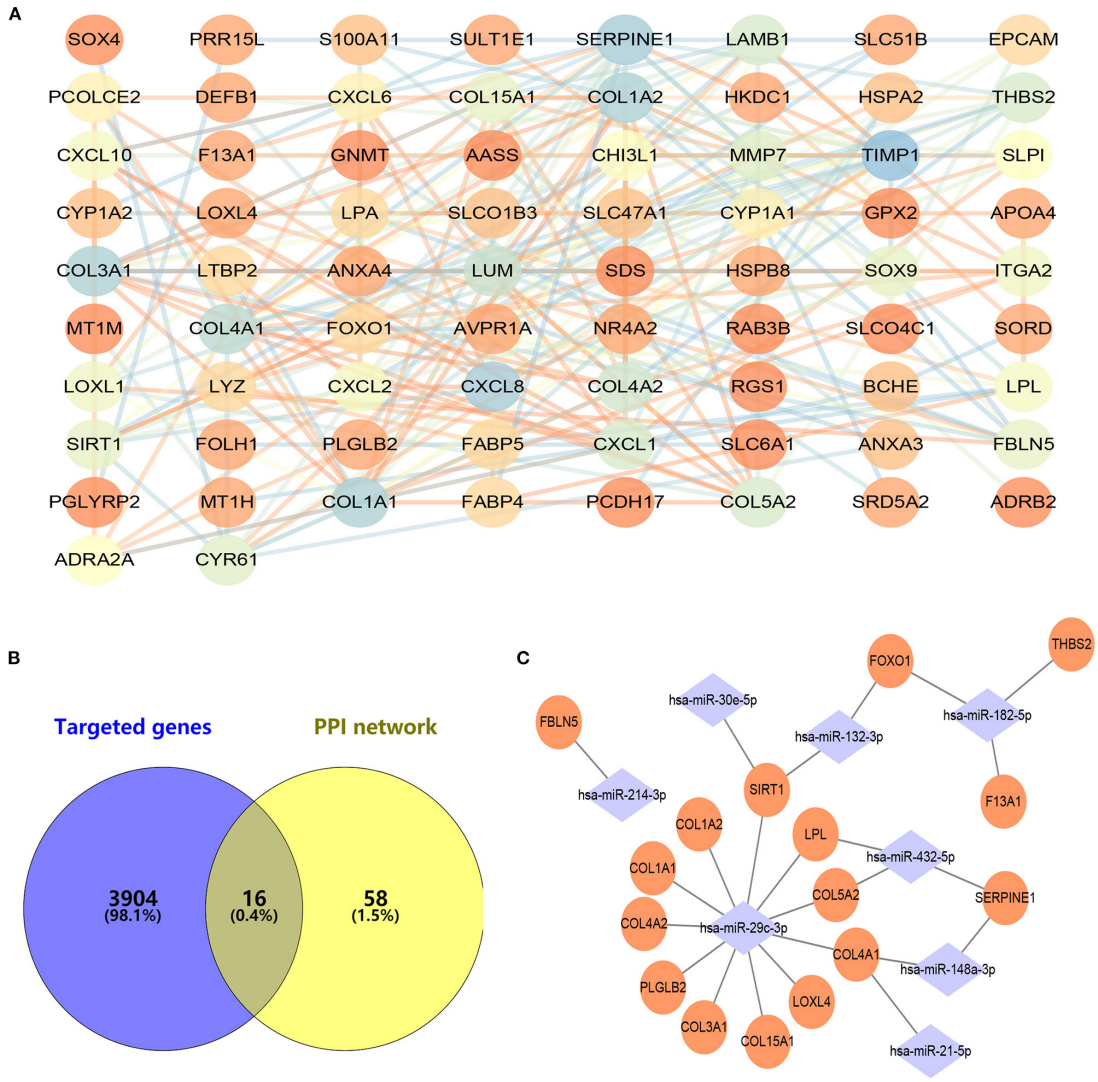
Construction of protein–protein interaction and miRNA–mRNA networks. **(A)** Protein–protein interaction of CEGs with 74 nodes and 207 edges, **(B)** exploration of hub mRNAs in ALD development, **(C)** establishment of an miRNA–mRNA network by using 16 hub genes and eight miRNAs. CEGs, common differentially expressed genes; ALD, alcoholic liver disease; PPI, protein–protein interaction.

With the STRING online tool, a total of 74 nodes and 207 edges were identified to be highly connected in the PPI network (Figure 3A). Target genes of DEMs were predicted with two independent databases, and only common genes were retained for subsequent analyses (Figure 3B). Afterwards, the miRNA–mRNA network was established to reveal the potential molecular mechanisms of ALD, including 16 common genes and eight miRNAs (Figure 3C). Of note, FOXO1 has been demonstrated to participate in lipid metabolism in our previous study and other publications (14–17). However, the expression of miR-182-5p between ALD tissues and normal liver tissues has remained controversial, and its molecular mechanisms were unclear (11, 12). Therefore, we reasoned that the hypothesis that miR-182-5p plays an essential role in ALD development by targeting FOXO1 was worthy of study in depth.

### Verification of Differentially Expressed Levels of miR-182-5p and FOXO1

**Figure 4:**
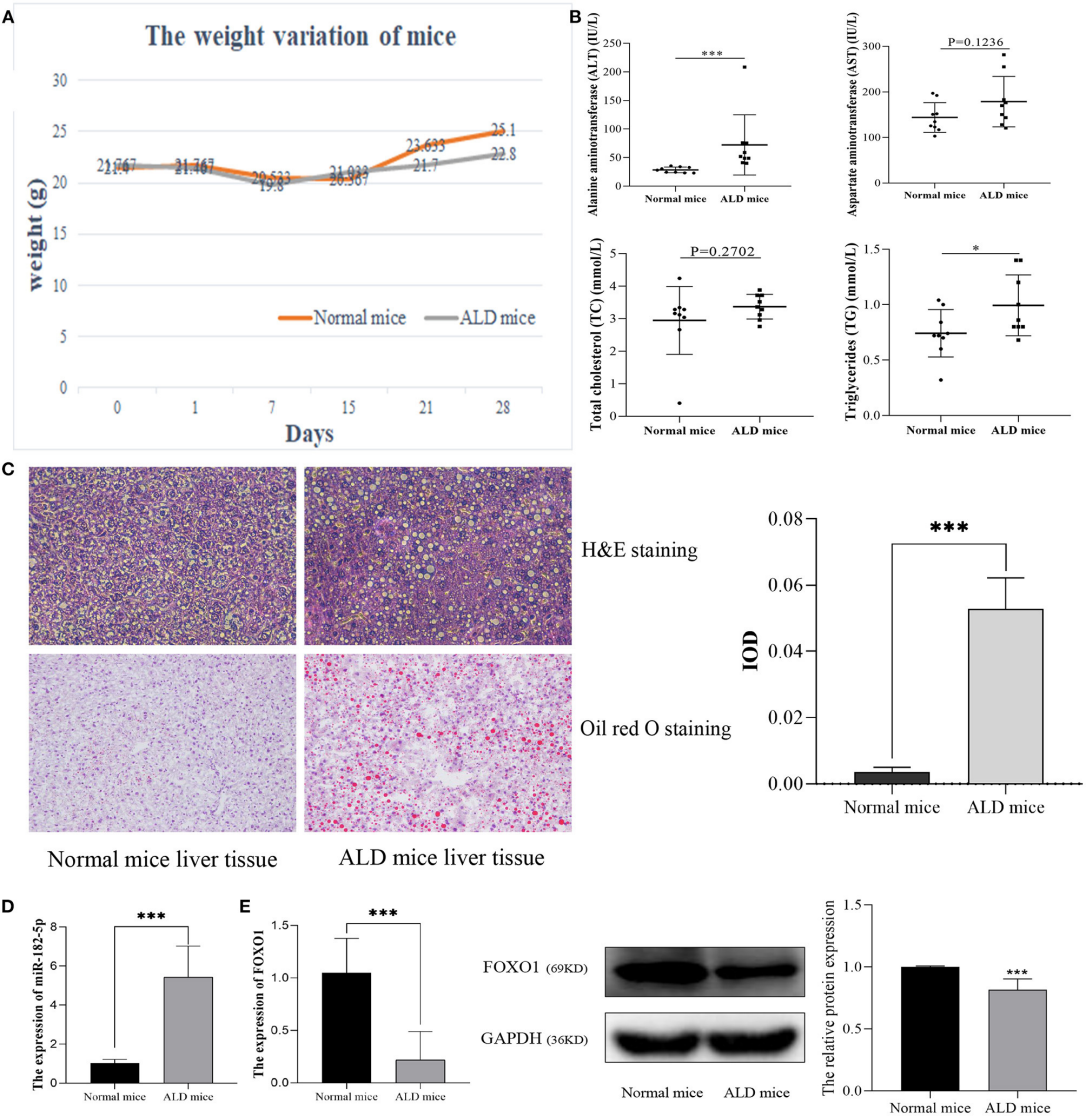
Validation of the expression of miR-182-5p and FOXO1 in the ALD mice model. **(A)** Weight variation in ALD mice, **(B)** biochemical characteristics of ALD mice, **(C)** liver tissue H&E, Oil Red O staining (× 40), and IOD analysis, **(D)** expression of miR-182-5p between ALD mice and normal mice, **(E)** quantitative real-time PCR and western blotting results of FOXO1 in ALD mice, respectively. ALD, alcoholic liver disease; ALT, alanine aminotransferase; AST, aspartate aminotransferase; TG, triglycerides; TC, total cholesterol. *p < 0.05, ***p < 0.001.

After being fed a Lieber-DeCarli diet for 28 days, ALD mice markedly differed from normal mice in their weight and biochemical characteristics. The statistical differences in weight variation between EtOH-fed and normal mice are shown in Figure 4A. Moreover, ALT and TG levels in EtOH-fed mice were markedly higher than those in normal mice. However, no significant difference was observed in TC and AST levels (Figure 4B). H&E staining and Oil Red O staining (Figure 4C) demonstrated that EtOH consumption significantly stimulated hepatic steatosis and accelerated fat accumulation in mice. Therefore, in this work, the ALD mouse model was successfully constructed. RT-qPCR was used to evaluate the relative expression levels of miR-182-5p and FOXO1 between ALD and normal liver tissues. The expression of miR-182-5p was dramatically up-regulated in ALD mice, whereas that of FOXO1 was lower, as compared with the levels in normal mice (Figures 4D,E).

**Figure 5:**
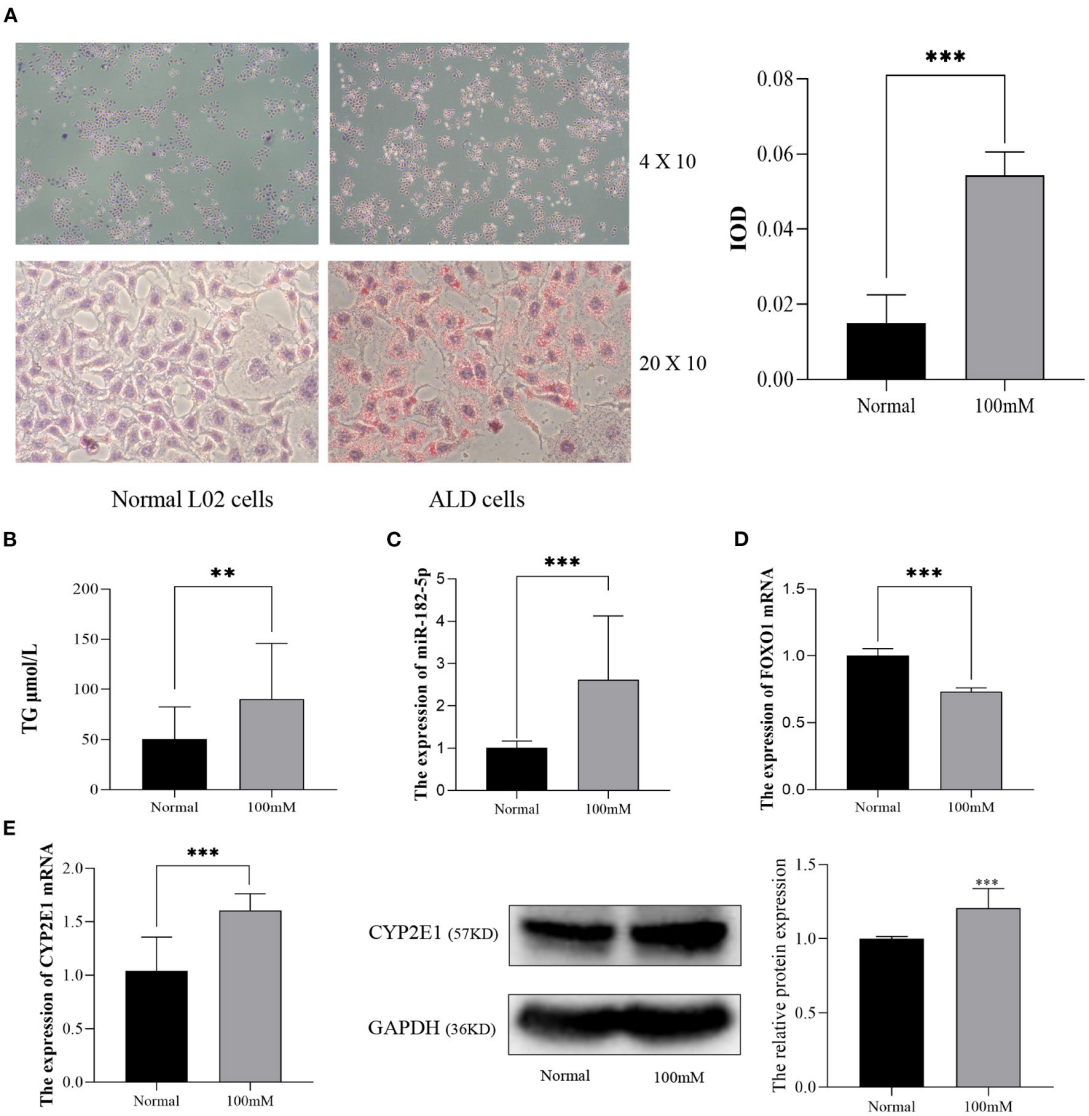
Validation of the expression of miR-182-5p and FOXO1 in an ALD cell model. **(A)** Oil Red O staining (× 40 and × 200) and IOD analysis of L02 cells with 100 mM alcohol, **(B)** difference in TG levels between ALD and L02 cells, **(C,D)** expression of miR-182-5p and FOXO1 mRNA, **(E)** expression of CYP2E1 between ALD and L02 cells. ALD, alcoholic liver disease; TG, triglycerides; IOD, integrated optical density. **p < 0.01, ***p < 0.001.

Next, L02 cells were exposed to 100 mM alcohol medium for 48 h, as described above, to establish the ALD cell model. As shown in Figure 5A, the cellular Oil Red O staining revealed that the lipid accumulation in ALD cells was much higher than that in normal L02 cells. Quantitative analysis indicated that the TG content and IOD of L02 cells dramatically increased as the EtOH stimulus was presented (Figure 5B). The differential expression of miR-182-5p and FOXO1 between ALD cells and L02 cells was consistent with the results in the mice model, thus further validating the RNA-seq expression chip results (Figures 5C,D). CYP2E1 expression was remarkably increased in ALD cell compared to normal liver cells (Figure 5E), suggesting that the alcohol metabolism ability of L02 cell was enhanced under EtOH stimulation.

### The Targeting Relationship Between miR-182-5p and FOXO1

**Figure 6:**
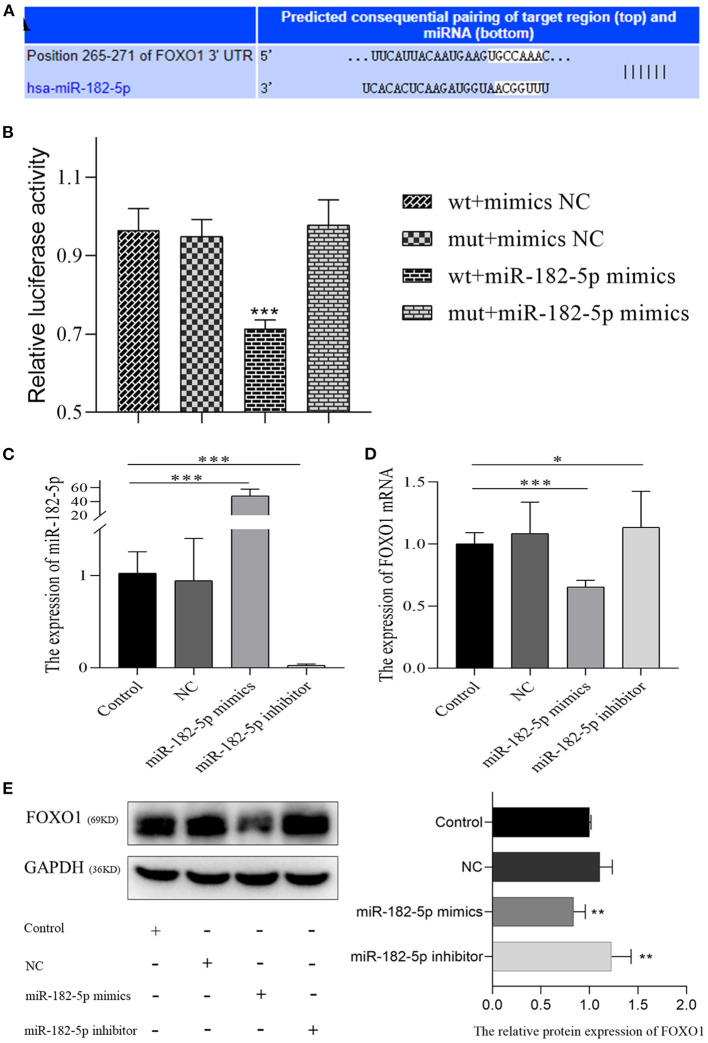
MiR-182-5p directly inhibits the expression of FOXO1. **(A)** The binding sites of MiR-182-5p targeting the 3'UTR of FOXO1 mRNA; **(B)** dual-luciferase reporter assay to confirm the targeting relationship between miR-182-5p and FOXO1; **(C)** verification of the expression of miR-182-5p with mimics, NC, and inhibitor transfection; **(D,E)** expression of FOXO1 mRNA and protein after transfection with miR-182-5p mimics NC, and inhibitor. NC, negative control; IOD, integrated optical density. *p < 0.05, **p < 0.01, ***p < 0.001.

According to TargetScan and miRDB databases, miR-182-5p binding to the 3'UTR of FOXO1 was identified (Figure 6A). Dual-luciferase reporter assays were performed to verify the targeting interaction. As shown in Figure 6B, the luciferase activity of the wild-type group that contained FOXO1 specific binding sites was clearly inhibited by co-transfection of miR-182-5p mimics. Importantly, the expression of FOXO1 mRNA was suppressed by over-expression of miR-182-5p (Figures 6C,D). Moreover, western blotting assays indicated that FOXO1 protein expression was markedly decreased in L02 cells transfected with miR-182-5p mimics (Figure 6E). In contrast, FOXO1 expression was positively up-regulated after miR-182-5p knockdown. Therefore, the above results demonstrated that miR-182-5p directly targets FOXO1 and inhibits its mRNA and protein expression.

### The Potential Molecular Mechanism of miR-182-5p Targeting FOXO1 in ALD Lipid Accumulation

**Figure 7:**
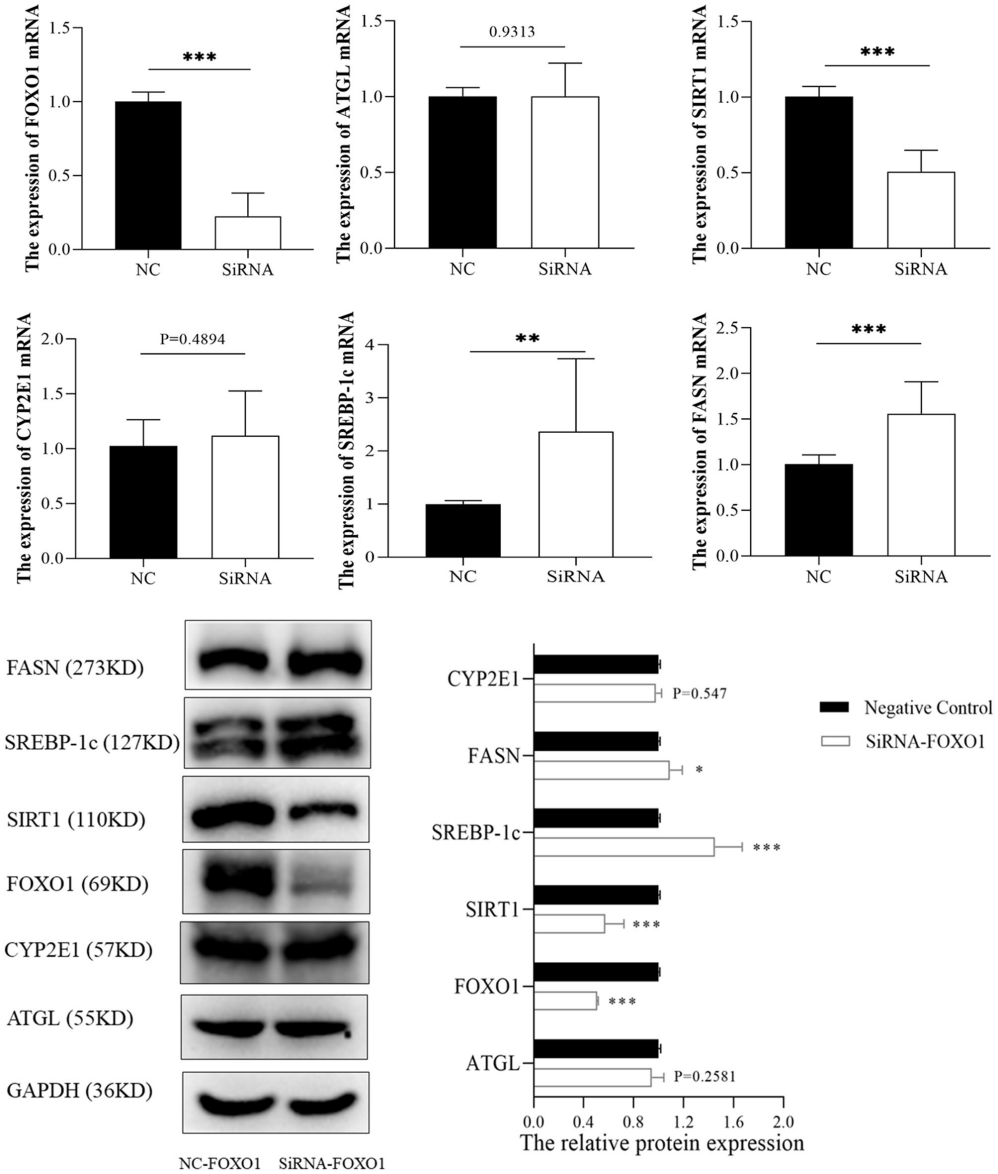
The expression of FOXO1 downstream genes between siRNA and negative control groups. *p < 0.05, **p < 0.01, ***p < 0.001. NC, negative control.

FOXO1, a hub transcription factor has been reported to have a critical function in fatty liver by regulating lipid metabolism-related gene expression. Hence, several lipid metabolism-associated downstream genes of FOXO1 previously confirmed by experimental studies (SIRT1, ATGL, SREBP-1c, and FASN) were detected to reveal the mechanism underlying the effects of FOXO1 in ALD. RT-PCR and western blotting assays indicated that the expression of FOXO1 was significantly decreased after transfection with FOXO1 siRNA (Figure 7). Further experiments showed that inhibiting FOXO1 in L02 cells promotes SREBP-1c and FASN, but represses SIRT1 expression. In comparison, ATGL and CYP2E1 expression levels were found no significant difference between siRNA and NC groups (*P* > 0.05).

**Figure 8:**
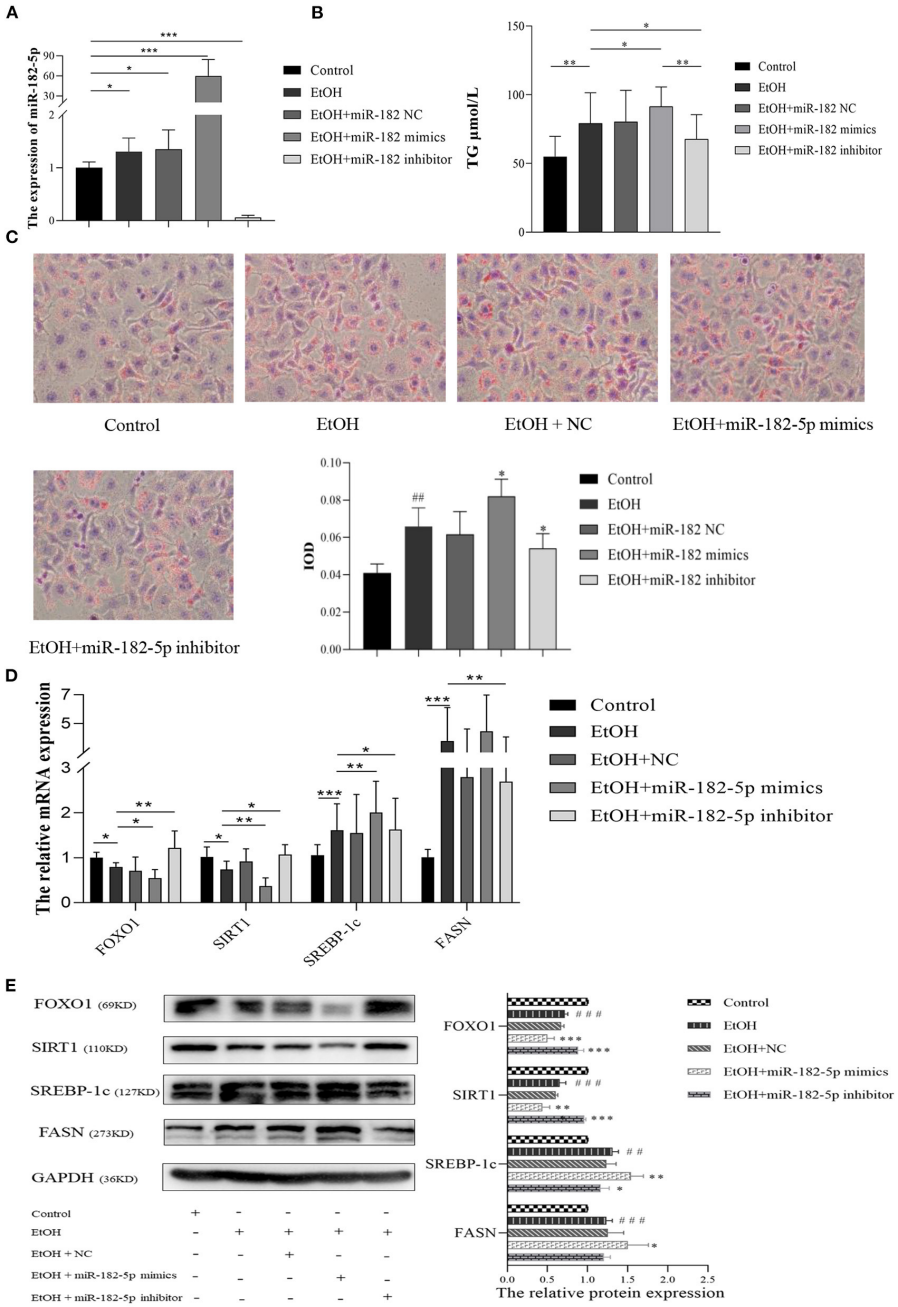
The molecular mechanism of miR-182-5p in ALD lipid accumulation. **(A)** Verification of miR-182-5p expression, **(B)** TG levels of ALD cells with miR-182-5p mimics, NC, and inhibitor transfection, **(C)** Oil Red O staining and IOD analysis, **(D)** mRNA expression levels of FOXO1 downstream genes, **(E)** western analysis of FOXO1, SIRT1, SREBP-1, and FASN protein expression after transfection. TG, triglycerides; NC, negative control; IOD, integrated optical density. ^##^*p* < 0.01, ^###^*p* < 0.001 vs. control group; *p < 0.05, **p < 0.01, ***p < 0.001 vs. EtOH group.

**Figure 9:**
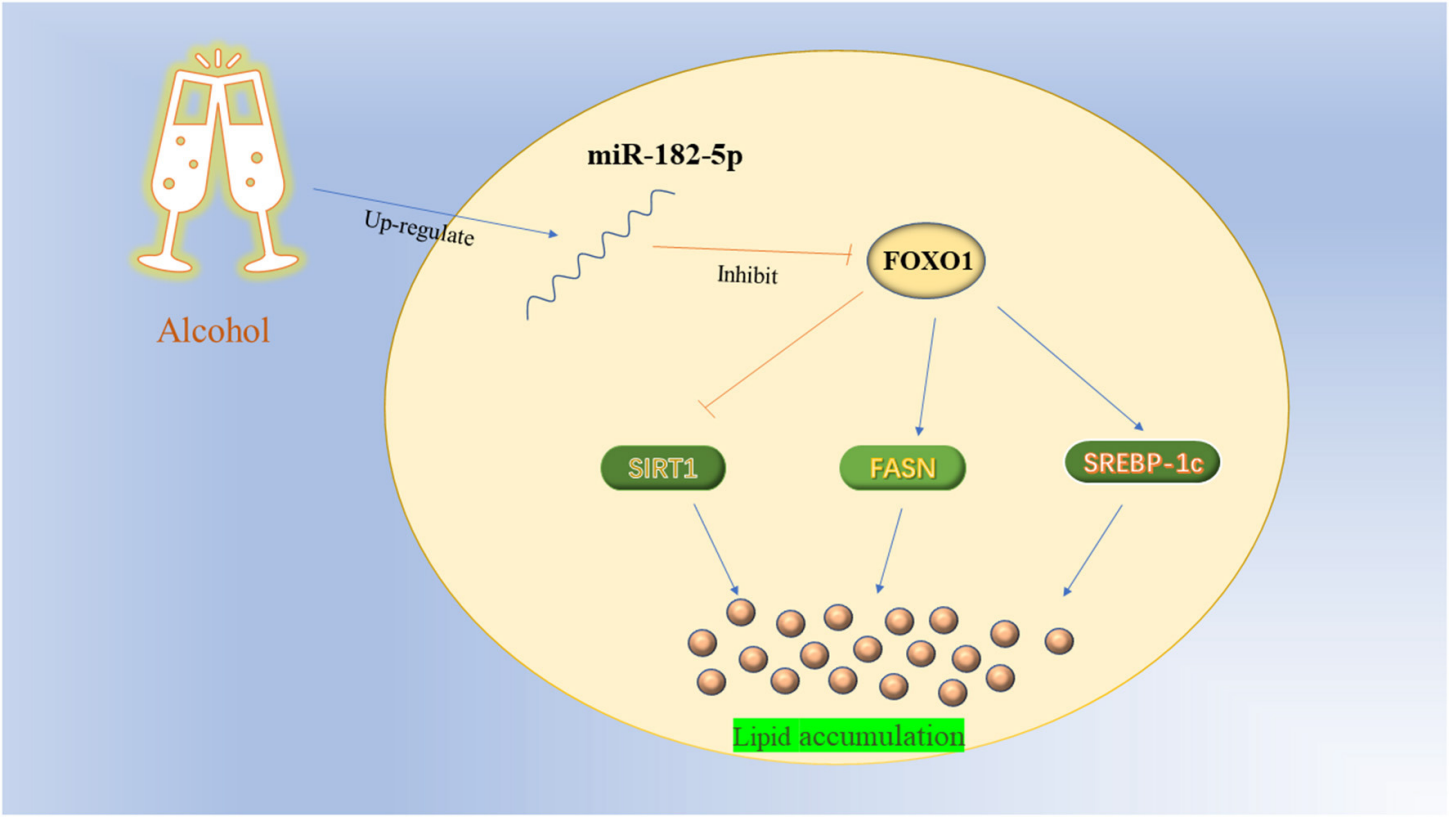
The working mechanism diagram of miR-182-5p/FOXO1 axis.

To further investigate the potential mechanism of miR-182-5p in ALD, we performed miR-182-5p overexpression and knockdown experiments. Quantitative real-time PCR showed that miR-182-5p expression in the mimic group was significantly higher than that in the ALD group, whereas an opposite trend in the knockdown group was observed, thus suggesting successful transfection in this work (Figure 8A). Interestingly, the TG level in the ALD model significantly decreased with miR-182-5p inhibitor treatment, whereas transfection of miR-182-5p mimics accelerated TG accumulation (Figure 8B). Moreover, Oil Red O staining and IOD analysis demonstrated that miR-182-5p knockdown markedly attenuated EtOH-induced hepatic steatosis (Figure 8C). Together, the above results revealed that miR-182-5p plays an essential role in TG deposition in ALD. As shown in Figures 8D,E, the expression of FOXO1 in ALD cells was clearly suppressed by EtOH exposure and upregulated miR-182-5p, but this effect was alleviated by transfection with miR-182-5p inhibitor. Meanwhile, SIRT1 expression was inhibited, whereas the expression of SREBP-1c, and FASN was greater in ALD than in the controls, thus verifying that an EtOH stimulus led to imbalanced lipid metabolism. Additionally, enhancement of the miR-182-5p/FOXO1 signaling axis dramatically stimulated SREBP-1c and FASN overexpression while repressing the expression of SIRT1. In contrast, its inhibition had opposite effects on ALD. These results demonstrated that the miR-182-5p/FOXO1 axis participates in lipid metabolism in alcoholic liver by regulating the expression of key lipid biosynthesis and decomposition-related genes (Figure 9).

## Discussion

In recent years, heavy alcohol intake has been globally prevalent, thus causing annual increases in the number of patients with ALD and posing a severe social burden (18). Early ALD is characterized by hepatic steatosis and hepatitis, during which effective treatment can avoid further liver damage. Currently, the best approach is to reduce alcohol consumption, but doing so is difficult over the long term due to the decreasing adherence in most ALD patients. Therefore, to identify hub molecules and further explore the underlying mechanism in ALD will contribute to the development of precise and novel therapeutic strategies.

MicroRNAs are viewed as key regulators that efficiently coordinate multiple cellular pathways. Thus, they have been suggested to have powerful potential as novel therapeutic candidates in various diseases. Importantly, the development of applications in pharmacological drug delivery and preclinical toxicology are making substantial progress (19, 20). MiR-182-5p has been validated to have an essential role in liver-related diseases. MiR-182-5p is a high-priority miRNA in HCC, and is closely associated with early recurrence and overall survival in patients (21, 22). Numerous studies have shown that miR-182-5p is dramatically overexpressed in HCC and could enhance the ability of migration, invasion, adhesion and proliferation of HCC cells via repressing multiple targeting genes, such as FOXO3a (21), Hepatitis C virus p7 trans-regulated protein 3 (P7TP3) (23), and regulator of calcineurin 1 (RCAN1) (24). Furthermore, high-throughput sequencing has revealed that miR-182-5p expression is dramatically increased in fatty liver-related fibrosis (25, 26). More importantly, Sedgeman et al. have demonstrated that miR-182-5p inhibition improves the glucose-lowering effects and significantly decreases cholesterol levels in the liver, thus suggesting a promising therapeutic target for fatty liver (27, 28). To our knowledge, however, the expression level of miR-182-5p in ALD remains controversial, and its molecular mechanism has scarcely been reported. In this work, miR-182-5p expression was notably higher in ALD patients than normal controls, on the basis of RNA-seq expression profiling. Furthermore, RT-PCR results in ALD mice and L02 cells showed that miR-182-5p was significantly up-regulated by alcohol consumption, closely associated with ALD lipid accumulation.

In addition to exploring hub molecules, we identified the miR-182-5p/FOXO1 axis as a key pathway in ALD development through bioinformatics analysis. FOXO1 is a member of the FOXO family of crucial transcriptional regulators involved in cell proliferation, oxidative stress, autophagy, and energy metabolism; the family comprises four proteins (FOXO1/3a/4/6) in mammals (29). Different FOXO factors are activated depending on the cell type features and circumstances. For instance, in the liver, FOXO1 is mainly responsible for gluconeogenesis and hepatic lipid metabolism; FOXO3a has pleiotropic functions in antioxidant responses and autophagy, as well as HCC cells proliferation and apoptosis (30). Previous studies have shown that FOXO1 expression is suppressed by alcohol stimulation. Heo et al. has reported that FOXO1 is down-regulated in ALD, and facilitates TXNIP overexpression and NLRP3 inflammasome activation, which induces hepatocyte pyroptosis (31). Likewise, our previous work has illustrated that FOXO1 expression in ALD tissues is markedly lower than that in normal liver tissues (16). Nevertheless, the role of FOXO1 in ALD lipid metabolism still remains unclear. According to ALD mouse and cell models, we found that FOXO1 was significantly repressed by alcohol consumption, thus further confirming its importance in ALD development. We used dual-luciferase reporter assays to explore the regulatory mechanism, which showed that miR-182-5p targets the 3′UTR binding site of FOXO1 as also demonstrated in Soheilifar's study (32). RT-PCR and western blotting revealed that over-expression of miR-182-5p dramatically decreased the expression of FOXO1 mRNA and protein. Additionally, inhibition of the miR-182-5p/FOXO1 signaling axis markedly ameliorated hepatic TG deposition caused by alcohol exposure.

Dysregulated hepatic FOXO1 induces an imbalance in lipid homeostasis by regulating genes involved in *de novo* lipogenesis, fatty acid oxidation, and lipolysis at the transcriptional level (29). SIRT1, a nicotinamide adenine dinucleotide (NAD+, NADH)-dependent class III histone deacetylase, is a key player in the regulation of lipid metabolism and the oxidative stress response (14). Accumulating data indicate that SIRT1 deacetylates lysine residues in the FOXO1 DNA binding domain and increases nuclear retention of FOXO1 (33). Importantly, they serve as the core genes in the FOXO/SIRT1 signaling pathway, and show close positive interactions in cells (33, 34). Previous studies have shown that SIRT1 is significantly inhibited by alcohol consumption, in agreement with our assay results (35). Adipose triglyceride lipase is a crucial regulator involved in fat catabolism. Emerging evidence indicates that FOXO1 markedly up-regulates the expression of ATGL, thus promoting lipolysis (36). Interestingly, Zhang et al. (37) have reported that this promoting effect may be mediated through suppressing the expression of the G0/G1 switch-2 gene, which encodes an inhibitor of adipose triacylglycerol lipase. Nevertheless, no significant difference in ATGL expression between FOXO1 inhibitor group and NC group was found in this work. Therefore, further research is required to investigate the regulatory relationship between FOXO1 and ATGL. In addition to its role in fat catabolism, FOXO1 participates in regulating many downstream target genes involved in lipid biosynthesis, such as FASN and SREBP-1c. Fatty acid synthase and SREBP-1c are important transcription factors that directly modulate the activity of key enzymes involved in cholesterol and fatty acid synthesis in cells (38, 39). Constant alcohol exposure promotes their expression, as also confirmed in this experiment, and increases the enzyme activity, thereby accelerating lipogenesis and lipid accumulation. Studies have indicated that FOXO1 decreases the transcription of FASN and SREBP-1c via combined actions on multiple transcription factors (40–42). Current evidence demonstrated that knockout of the miR-182-5p/FOXO1 axis significantly induced expression of FASN and SREBP-1c genes, and increased SIRT1 expression, thereby ameliorating excessive lipid accumulation in ALD cells.

The present work represented the first application of bioinformatics analysis and experimental studies that primarily aimed to identify hub molecules and explore the underlying signaling pathway for ALD development. Importantly, RNA-seq expression profiling and mouse and cell models were used to verify the differential expression levels. In addition, we also investigated the mechanism underlying the effects of miR-182-5p in ALD. However, several limitations with our study remained. Firstly, the induction of FOXO1 in miR-182-5p inhibitor group was modest compared to the control group, which may resulted from the low expression of miR-182-5p in normal cell and the mutual regulation of other pathways. Secondly, further in-deep experiments and clinical studies are still required to confirm the potential of miR-182-5p as a therapeutic target for ALD.

## Conclusions

In summary, key molecules were identified and a comprehensive miRNA–mRNA network was established to reveal the potential pathways for ALD though RNA-seq expression profiles. Moreover, the miR-182-5p/FOXO1 signaling axis was identified as a crucial pathway in lipid metabolism in ALD. Importantly, our results suggested that miR-182-5p in liver cells is significantly increased by alcohol consumption, and its overexpression promotes hepatic lipid accumulation by targeting the FOXO1 signaling pathway. Our findings provided novel scientific insights and potential therapeutic targets for ALD.

## Data Availability Statement

The original contributions presented in the study are included in the article/Supplementary Material, further inquiries can be directed to the corresponding author/s.

## Ethics Statement

The animal study was reviewed and approved by the Ethics Committees of Southwest Medical University.

## Author Contributions

ZZ and YL performed the experiment, data analysis, charting, and writing—original draft this article. CZ worked on design and supervision of review. YX contributed to data correction and formal analysis. HT and YG worked on design and supervision of review, funding acquisition, and project administration. All authors have read and agreed to the published version of the manuscript.

## Funding

This study was supported by Luzhou Municipal People's Government and Southwest Medical University (Grant No. 2018LZXNYD-ZK08), Applied Basic Research Foundation of Sichuan Provincial Science and Technology Department (No. 2021JY0240), and Sichuan Provincial Health Commission (Grant No. 20PJ144).

## Conflict of Interest

The authors declare that the research was conducted in the absence of any commercial or financial relationships that could be construed as a potential conflict of interest.

## Publisher's Note

All claims expressed in this article are solely those of the authors and do not necessarily represent those of their affiliated organizations, or those of the publisher, the editors and the reviewers. Any product that may be evaluated in this article, or claim that may be made by its manufacturer, is not guaranteed or endorsed by the publisher.
