# Supplementary figures and images for: Integrated Analyses Identify Key Molecules and Reveal the Potential Mechanism of miR-182-5p/FOXO1 Axis in Alcoholic Liver Disease
Source: Front Med (Lausanne). 2021 Dec 7;8:767584. doi: 10.3389/fmed.2021.767584 (PMC8688759; doi:10.3389/fmed.2021.767584)

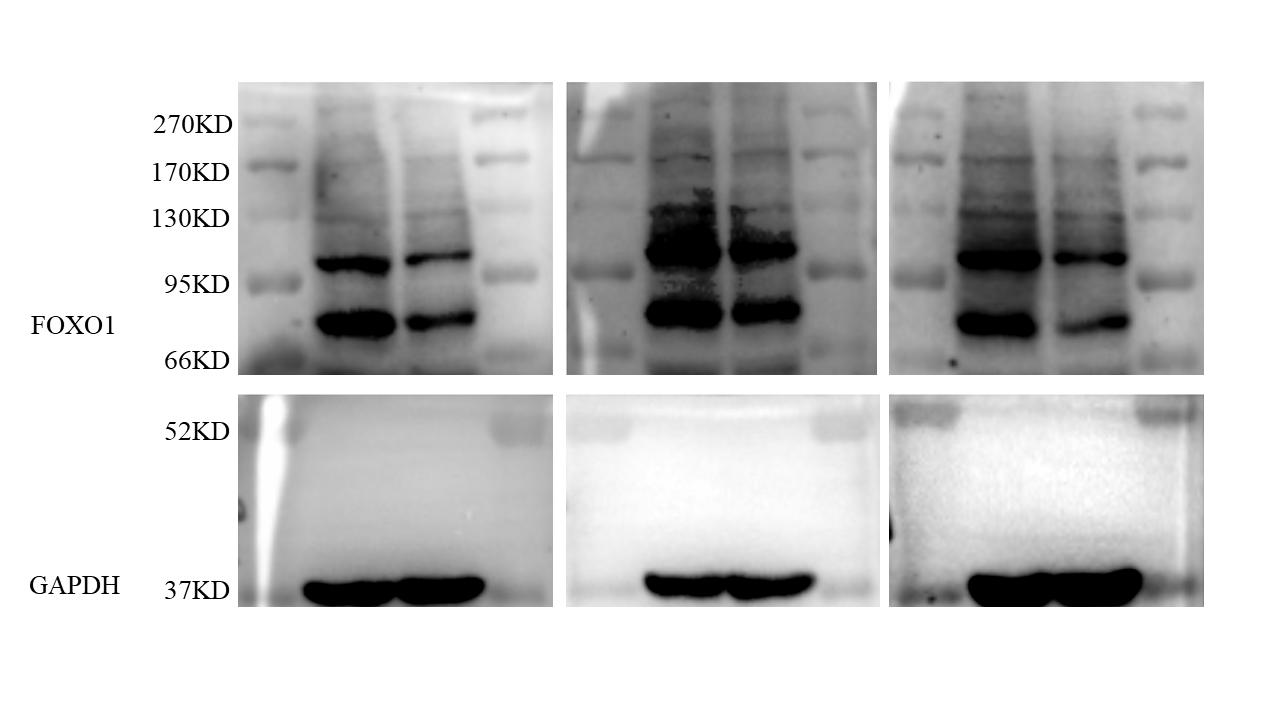

Supplement: Supplementary file 1 [file Data_Sheet_1.zip › Original data and figures/Figure 4.tif]

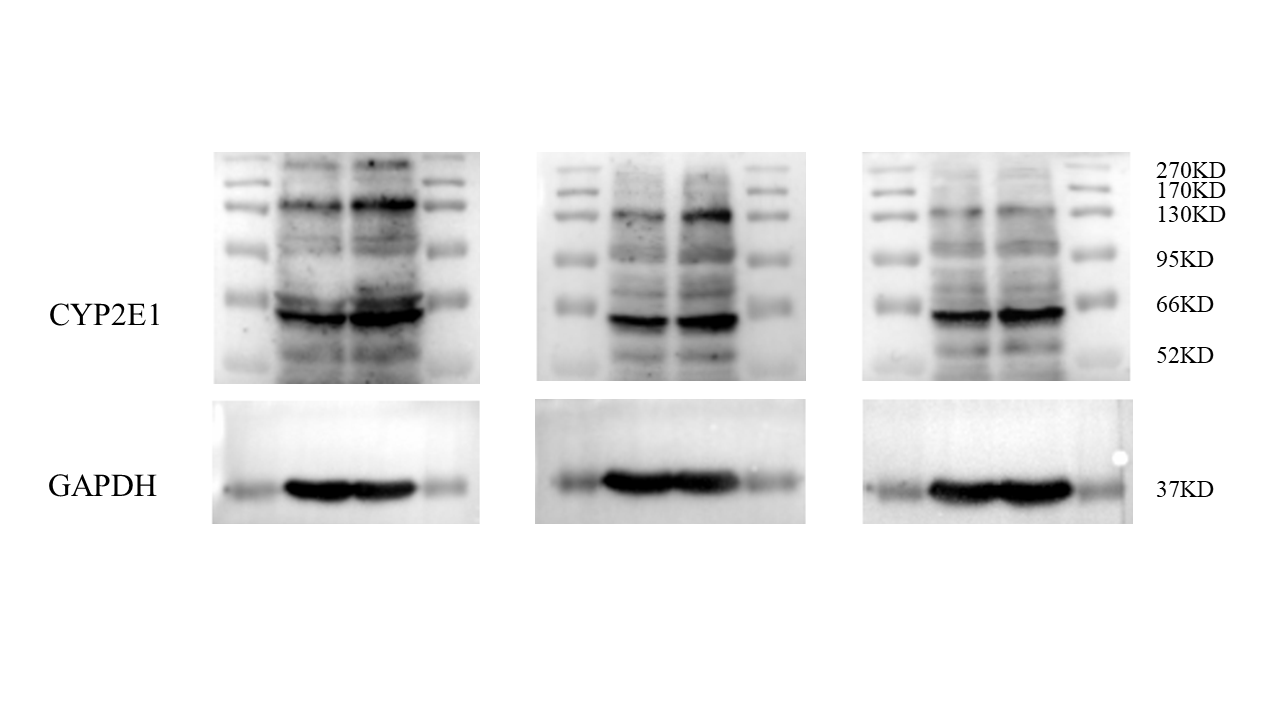

Supplement: Supplementary file 1 [file Data_Sheet_1.zip › Original data and figures/Figure 5.tif]

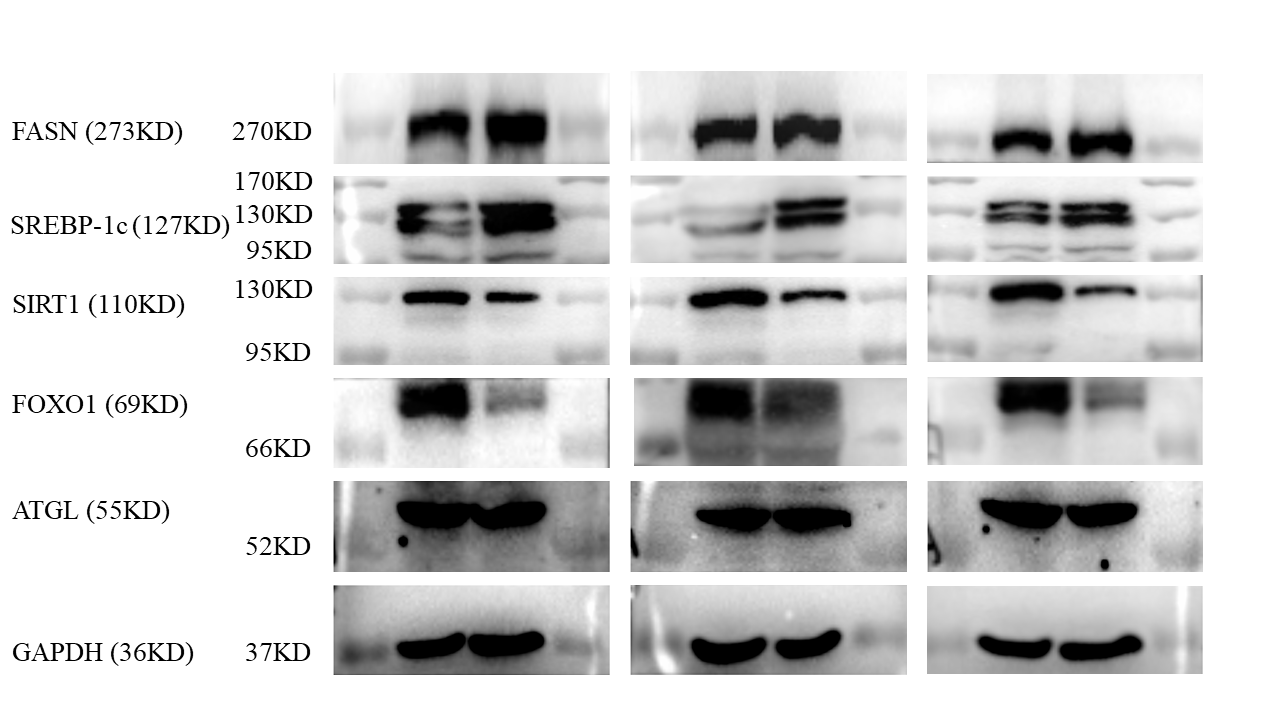

Supplement: Supplementary file 1 [file Data_Sheet_1.zip › Original data and figures/Figure 7-1.tif]

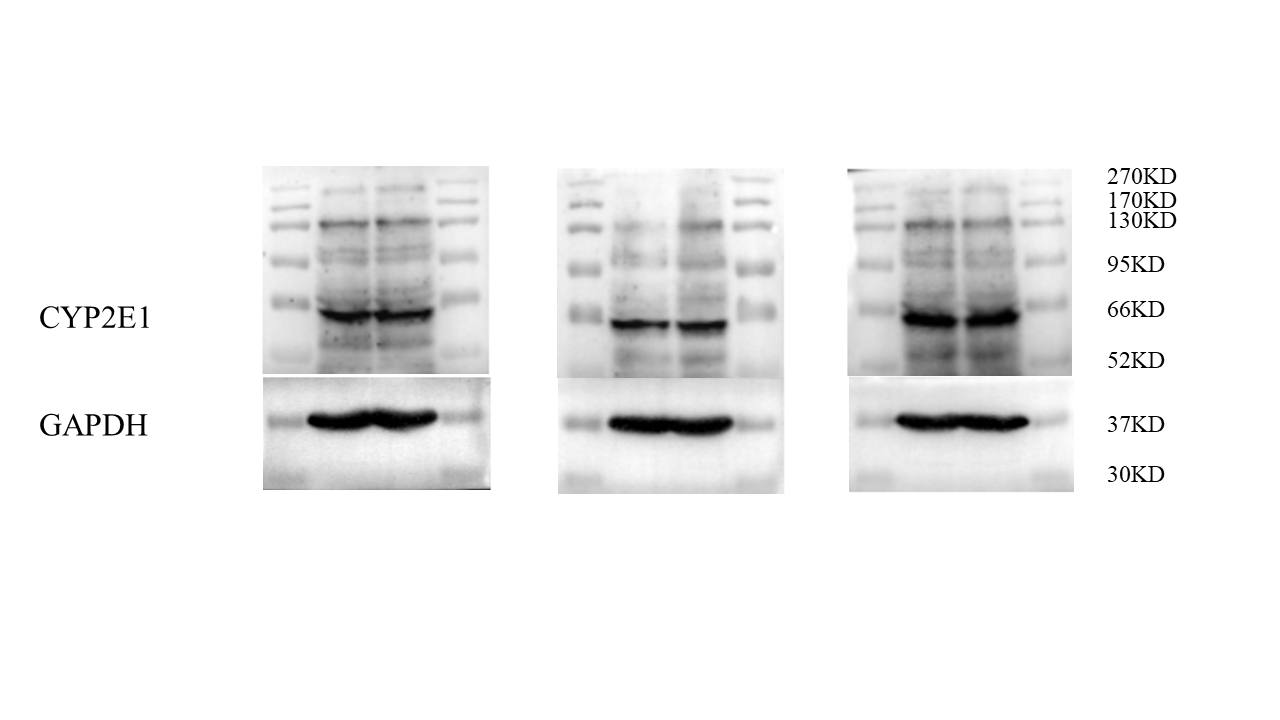

Supplement: Supplementary file 1 [file Data_Sheet_1.zip › Original data and figures/Figure 7-2.tif]

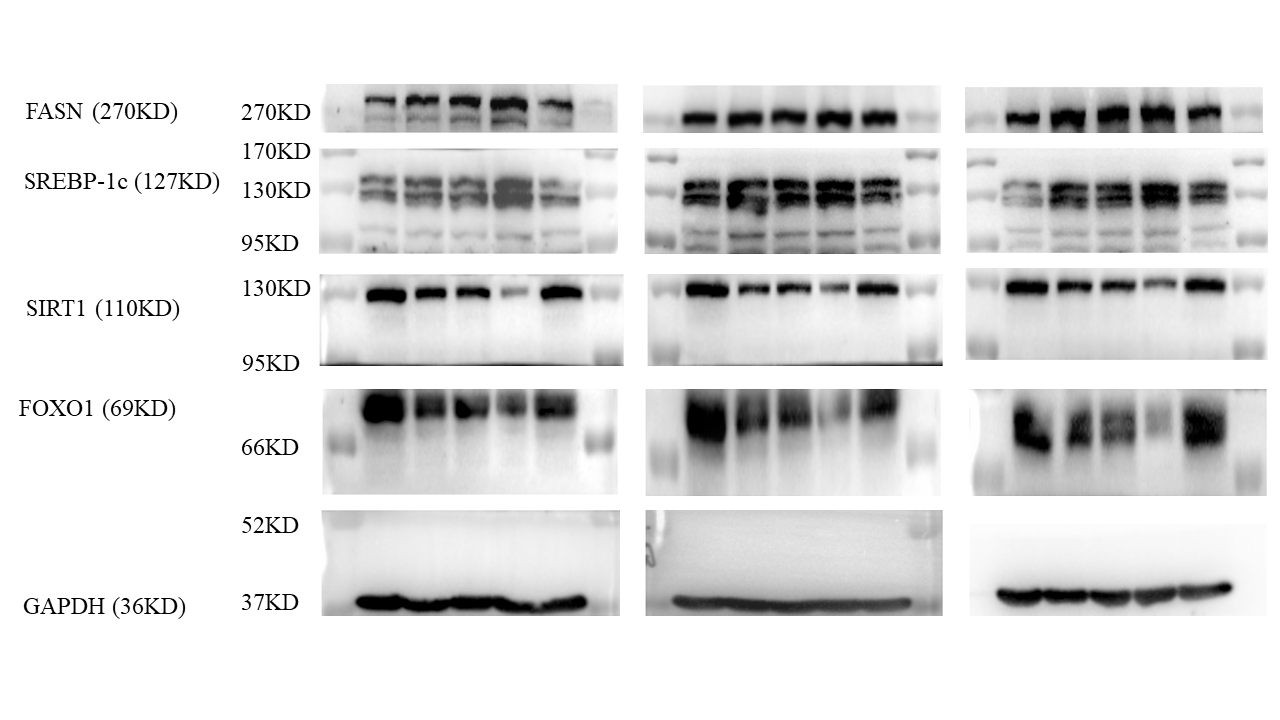

Supplement: Supplementary file 1 [file Data_Sheet_1.zip › Original data and figures/Figure 8.tif]

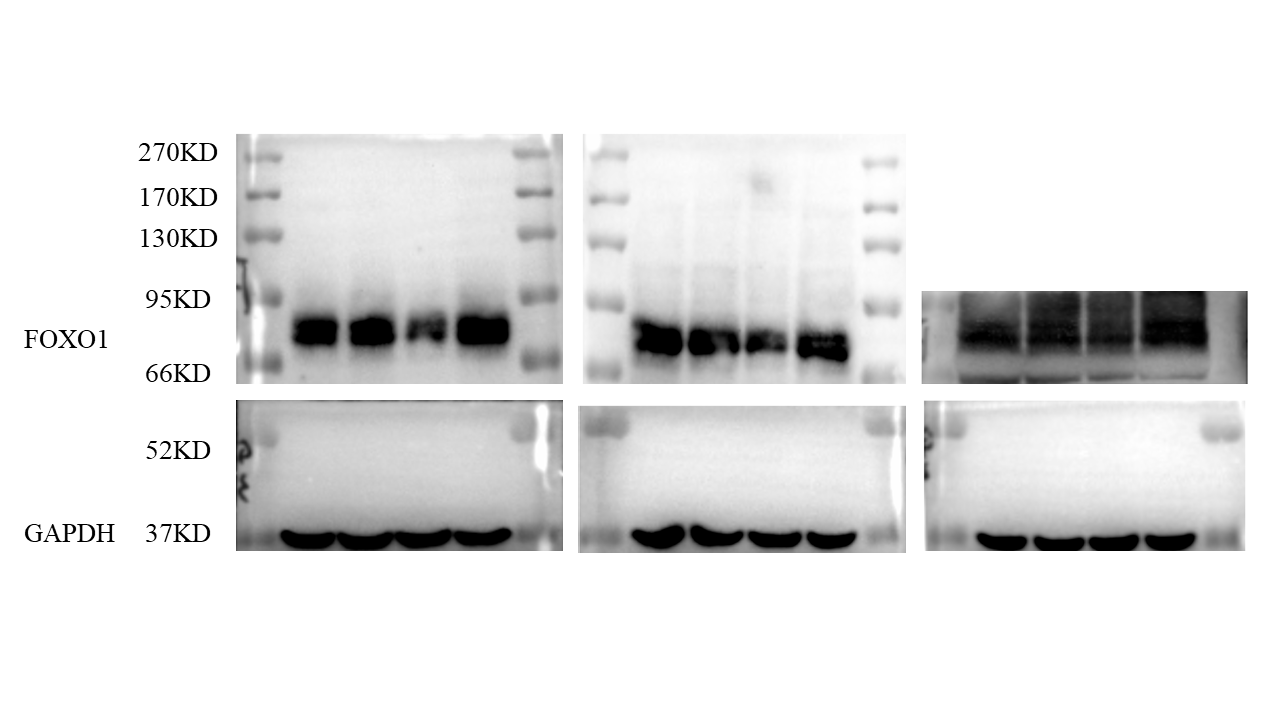

Supplement: Supplementary file 1 [file Data_Sheet_1.zip › Original data and figures/figure 6.tif]
